# The chaperonin TRiC component Cct3 is required for axonal transport, myelination, and neuromuscular junction refinement

**DOI:** 10.1038/s41419-026-08465-y

**Published:** 2026-02-12

**Authors:** Xiaomeng Zhang, Kamil Kajetan Zajt, Tayfun Palaz, Lisa Wang, Martin Groß, Florian Kraft, Joachim Weis, Juliane Bremer

**Affiliations:** 1 Institute of Neuropathology, Uniklinik RWTH Aachen, Aachen, Germany; 2 Center for Human Genetics and Genomic medicine, University Hospital RWTH Aachen, Aachen, Germany

**Keywords:** Developmental disorders, Experimental models of disease, Neurodevelopmental disorders

## Abstract

TRiCopathies are recently discovered neurodevelopmental diseases caused by pathogenic variants in components of the chaperonin tailless complex polypeptide 1 ring complex (TRiC). Composed of chaperonin containing TCP1 subunits 1-8 (CCT1-8), TRiC acts as a chaperone that is required for folding of 10% of proteome, including actin and tubulin. Patients with TRiCopathies display variable combinations of cognitive impairment, epilepsy, polymicrogyria, white matter reduction, cerebellar hypoplasia and alterations in the peripheral neuromuscular system. Here, we aimed at better understanding the pathophysiological role of Cct3 in neurodevelopment, particularly in myelin formation and the neuromuscular system using zebrafish as a model system. We have generated two CRISPR/Cas9 loss-of-function alleles of the orthologous zebrafish *cct3* gene. By combining these alleles with transgenic lines and immunostainings we visualized different cell types and subcellular structures in the nervous system by confocal microscopy. Furthermore, we performed electron microscopy to examine zebrafish in comparison to human patient-derived tissue. We demonstrated that *cct3* mutant zebrafish fail to form normal myelin sheaths. This was associated with early apoptotic death of neural crest-derived Schwann cells, which were particularly vulnerable to loss of Cct3 function. In line with the observations in patients, developmental refinement of neuromuscular junctions (NMJ) required Cct3. Furthermore, we observed profound cytoskeletal alterations, in particular of tubulin and microtubules as well as severely disturbed microtubule-dependent axonal transport of organelles in peripheral motor axons. Cct3 displays an essential role in myelination, survival of neural crest-derived cells, NMJ refinement, tubulin and microtubule biology as well as axonal transport. Given that axonal transport is essential for transport of axon-glial and signaling factors shaping the NMJ, we speculate that the essential role of Cct3 in axonal transport is a common denominator for the observed phenotypes. These data enhance our understanding of the conserved role of Cct3/TRiC in the developing nervous system and the pathophysiological mechanisms in TRiCopathies.

## Introduction

Pathogenic variants in components of the chaperonin tailless complex polypeptide 1 ring complex (TRiC) were recently identified as a cause of a group of developmental diseases of the nervous system termed “TRiCopathies” [1]. TRiC belongs to the group of molecular chaperones and forms a ring-like oligomer that assembles into a cylindrical complex upon dimerization, creating a cavity with substrate-binding sites that protect the substrates from the environment and facilitate the ATP-dependent folding of proteins [2]. TRiC is a 1 MDa hexadecamer that comprises two antiparallel rings of eight paralogous subunits called Chaperonin Containing TCP1 Subunit (CCT) 1-8, also called CCT*α*, *β*, *γ*, *δ*, ε, *ζ*, *η*, *θ* [3]. The complex is responsible for folding of ~10% of the proteins of the proteome [4], including actin and tubulin [5, 6]. In all three patients with pathogenic variants in *CCT3* (Q396Kfs*27, two with R518*) with available brain MRI, a reduction of the white matter was observed [1], suggesting a role of *CCT3* in myelination or myelin maintenance. Similarly, in patients with *CCT1* and *CCT5* mutations, imaging results suggest myelin involvement in at least some cases [1].

Major cytoskeletal components, i.e. actin filaments and microtubules are folded by TRiC. The dynamics of both components are essential for different aspects of cell migration and shaping processes, including intracellular transport, axon guidance and myelination [7, 8]. Precursor cells of myelinating cells, especially neural crest-derived cells, that develop into Schwann cells and many other cell types, migrate intensively before they differentiate into their derivatives [9]. Later in differentiation, when oligodendrocytes and Schwann cells need to expand their processes for myelination, they require modulation of actin filaments, which was demonstrated in mice lacking filament-regulating proteins such as WAVE1/Wasf1 in oligodendrocytes [10] or N-WASP/Wasl in Schwann cells [11, 12]. In oligodendrocytes initial process extension requires fast Arp2/3-driven F-actin assembly, whereas the process of myelin wrapping is characterized by rapid disassembly of F-actin controlled by myelin basic protein (Mbp) via phosphatidylinositol-4,5-bisphosphate (PIP2) signaling [13]. In mature oligodendrocytes and Schwann cells, microtubules are the main class of cytoskeletal elements. They form trafficking routes for vesicular transport as well as transport of *mbp* mRNA. Studies in zebrafish, identified tubulin alpha 8-like 3a (tuba8l3a) and tubulin-associated motor protein (kif1b) as essential for myelination [14, 15]. Combined, this evidence points at a crucial role of the cytoskeleton and therefore probably TRiC in myelinating cells. However, how TRiC controls myelination remains still to be unraveled. In addition to a primary defect in myelinating cells, axons are known to provide essential signaling molecules, regulating Schwann cell survival, myelination and myelin maintenance [16–18]. Hence, a primary axonal pathology with a secondary effect on glial cells is another possible explanation for the observed myelin reduction. In line with axonal disturbances, electron microscopy of muscle biopsies of the *CCT3* patient with the Q396Kfs*27 variant revealed accumulated organelles, i.e. autophagic vacuoles in intramuscular peripheral nerve fiber axons as well as a paucity of synaptic folds at axon endings [1], the latter also pointing at a role of TRiC/ cct3 in neuromuscular junction formation that is conserved in *Drosophila melanogaster* [19].

Using CRISPR/Cas9, we have generated two loss-of-function alleles of the orthologous zebrafish *cct3* gene. Similar to some patients, mutant fish show cerebellar hypoplasia as well as irregularities in F-actin distribution, demonstrating a conserved role of *cct3* in cerebellar development and F-actin proteostasis [1]. Pathogenic variants in *CCT2* had previously been identified in patients with congenital amaurosis, which could also be modeled in zebrafish [20, 21]. Mutants showed reduced number of photoreceptors and retinal ganglion cells (RGCs), increased RGC cell death and reduced retinotectal-projecting axons. Similar findings were observed in the developing eyes of *cct3* [22] and *cct5* zebrafish mutants, while in *cct4* mutants the eyes were severely degraded and only rudimentary structures of lens and pigmented retina were recognizable [23]. Altogether, these data suggest that also other cells are vulnerable to loss of TRiC components and that subunit-specific differences exist.

In the present paper, we are now focusing on the pathophysiology of the other important neurodevelopmental disturbances of patients with TRiCopathies, in particular, the reduction of the white matter and the abnormalities observed in neuromuscular junctions. Furthermore, we aimed at better understanding the consequences of loss of *cct3* function on its important substrate, the neuronal cytoskeleton. We found that *cct3* is dispensable for the outer appearance of motor neuronal axons during the first days of development, but essential for myelination at least in part by ensuring survival of neural crest-derived cells/Schwann cells along peripheral nerves. Furthermore, we show that the developmental refinement of neuromuscular junction structure requires *cct3* in zebrafish. Despite the normal outer appearance of motor axons, using electron microscopy and live imaging, we observed profound disturbances of the microtubule and actin cytoskeleton in zebrafish neurons. Axonal transport of organelles, i.e. mitochondria and Rab7+ endosomes, which strongly depends on an intact axonal cytoskeleton, especially on microtubules, also required *cct3*.

We demonstrate the significance of TRiC’s role in particular in Schwann cell survival, myelination as well as in tubulin and microtubule biology. Hence, our findings enhance our understanding of the conserved role of Cct3/TRiC in the developing nervous system and the pathophysiological mechanisms in TRiCopathies.

## Results

### Generation of zebrafish loss-of-function *cct3* mutant alleles

**Fig. 1:**
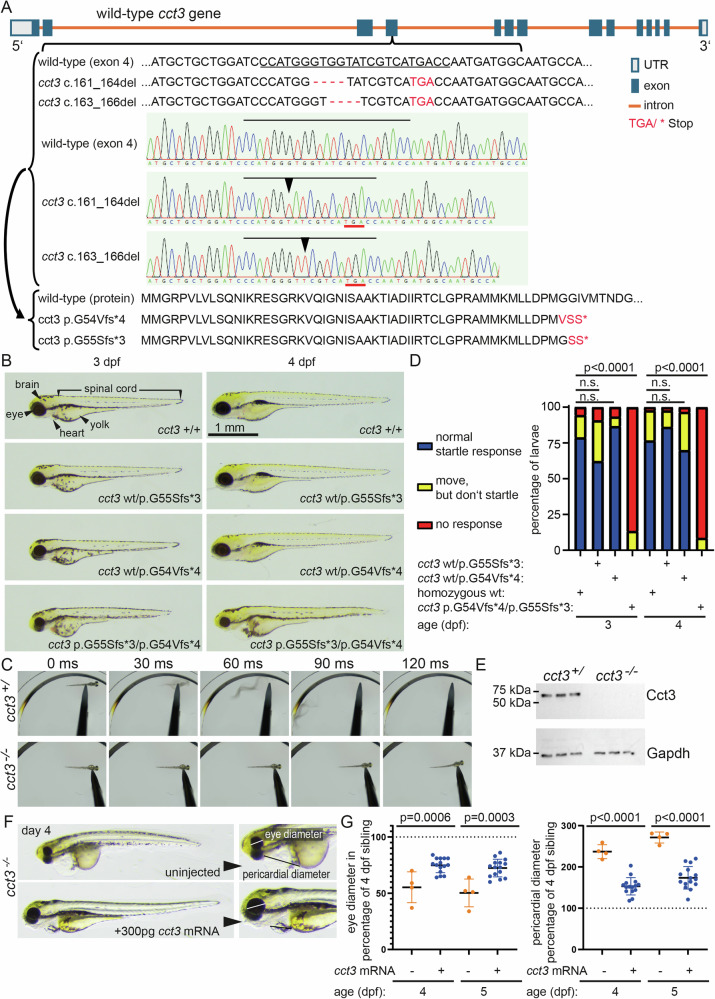
Zebrafish loss-of-function *cct3* mutant. CRISPR/Cas9 was used to generate loss-of-function alleles of *cct3*. Exon 4 was targeted (target sequence is underscored), two mutant alleles were selected that cause frame shift mutations, predicted to cause a premature stop and a truncated protein (**A**). Mutant larvae at 3 and 4 days of age are shown (**B**). They are smaller in overall size, in particular brain and eyes are smaller. This difference gets more pronounced with age. On day 4, mutant larvae also display cardial edema. Furthermore, mutant larvae have a reduced response to a touch stimulus. Different time points after stimulus are shown for 3-day-old sibling and mutant larvae (**C**), showing S-shaped body curvature and displacement of a sibling, but not of mutant larva. Quantification demonstrates significantly altered touch response at 3 and 4 dpf in *cct3* mutants, compared to wt and heterozygous siblings. *P* values were determined using Fisher’s exact test (**D**). Western blot shows undetectable Cct3 protein in mutants at 4 dpf (**E**). Injection of *cct3* mRNA partially rescues the mutant phenotypes. Eye diameter and pericardial edema were significantly improved, compared to uninjected mutants from the same clutches. Each data point corresponds to one fish at 4 and 5 dpf (**F**, **G**). *P* values were determined using two-tailed unpaired Student’s t tests.

We generated loss-of-function alleles of zebrafish *cct3* by targeting the 4th exon using CRISPR/Cas9 (Fig. 1). We selected two mutant alleles that caused frame shift mutations, predicted to result in a premature stop and a truncated protein product (Fig. 1A). Compared to homo- and heterozygous siblings (homozygous wild-type (wt), *cct3* wt/p.G55Sfs*3 heterozygous and *cct3* wt/p.G54Vfs*4 heterozygous), *cct3* compound heterozygous larvae (*cct3* p.G54Vfs*4/p.G55Sfs*3) displayed the mutant phenotype, characterized by smaller body length, smaller heads/brains and eyes, especially on day 3 and 4 of life, similar to homozygous mutants of the p.G54Vfs*4 allele, which we used for subsequent analysis throughout this manuscript (Supplementary Fig. 1). On day 4, mutant larvae also display cardiac edema (Fig. 1B). Circulation ceased and mutant larvae were not viable beyond day 5. While siblings readily responded with a startle response and displacement after touch stimulus, mutants showed significantly impaired response to touch stimulus (Fig. 1C, D). Cct3 protein was undetectable in mutant larvae at 4 dpf by Western blot (Fig. 1E, Supplementary Original data). To demonstrate that the mutant phenotype was indeed due to loss of Cct3, we reintroduced *cct3* mRNA by microinjections into one cell-stage embryos. Compared to uninjected *cct3* mutants, the phenotypes were significantly reduced in mutants from the same clutches that had received 300 pg of wt *cct3* mRNA 4 days prior to the analysis (Fig. 1F, G).

### *Cct3* is required for myelination, Schwann cell shaping, and survival of neural crest-derived cells

**Fig. 2:**
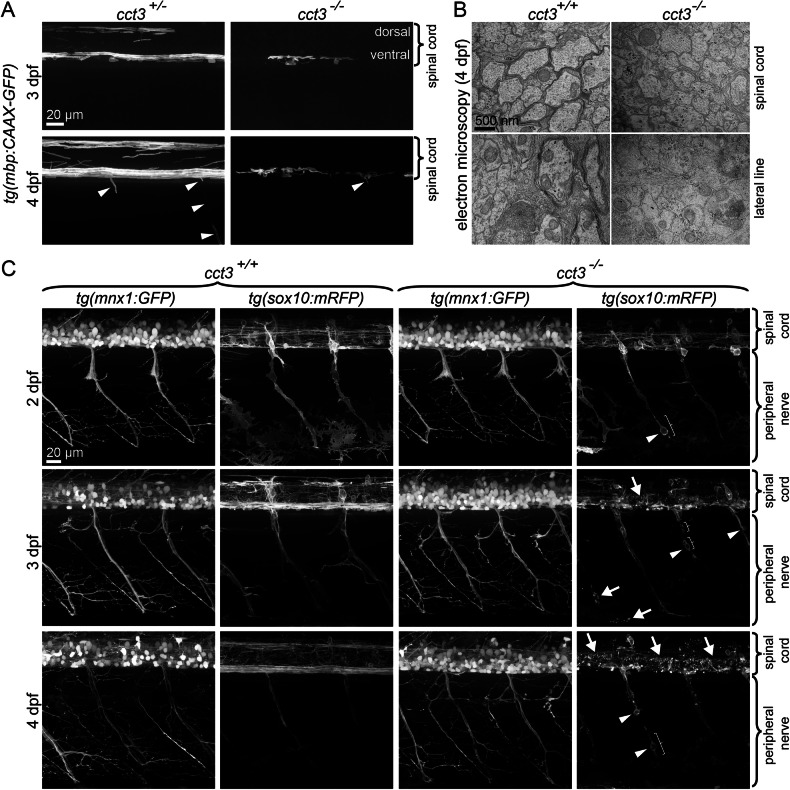
Cct3 is required for myelination. **A** Myelinating cells are activating the *mbp* promoter and are therefore labeled by membrane-bound GFP in *tg(mbp:EGFP-CAAX)*^*ue2*^ larvae. At 3 dpf, myelinating cells are mainly seen in the ventral spinal cord in *cct3* siblings with few myelin segments seen also in the dorsal spinal cord. At 4 dpf, more myelinating cells are seen in the ventral and dorsal spinal cord as well as along peripheral nerves (arrowheads) in siblings. In contrast, there are fewer myelinating segments in the ventral spinal cord of *cct3* mutants and very little labeling at the roots of peripheral nerves (arrowhead). **B** Electron microscopy shows several layers of developing myelin lamellae and incipient compaction around wt axons (left column) at 4 dpf in the spinal cord (upper row) as well as in the lateral line (lower row). Myelin sheaths are absent in *cct3* mutants (right column). **C** In contrast to myelinating cells, no obvious morphological differences are observed in motoneuronal axons labeled with GFP in *tg(mnx1:GFP)*^*ml2*^ transgenic fish between 2 and 4 dpf in *cct3* mutants compared to wt siblings. Schwann cells and their precursors labeled in *tg(sox10:mRFP)*^*vu234*^ transgenic fish often displayed a more rounded shape (arrowheads), shorter processes (curly braces) and cellular fragmentation, which was also seen in neural crest cells at the level of the spinal cord (white arrows).

**Fig. 3:**
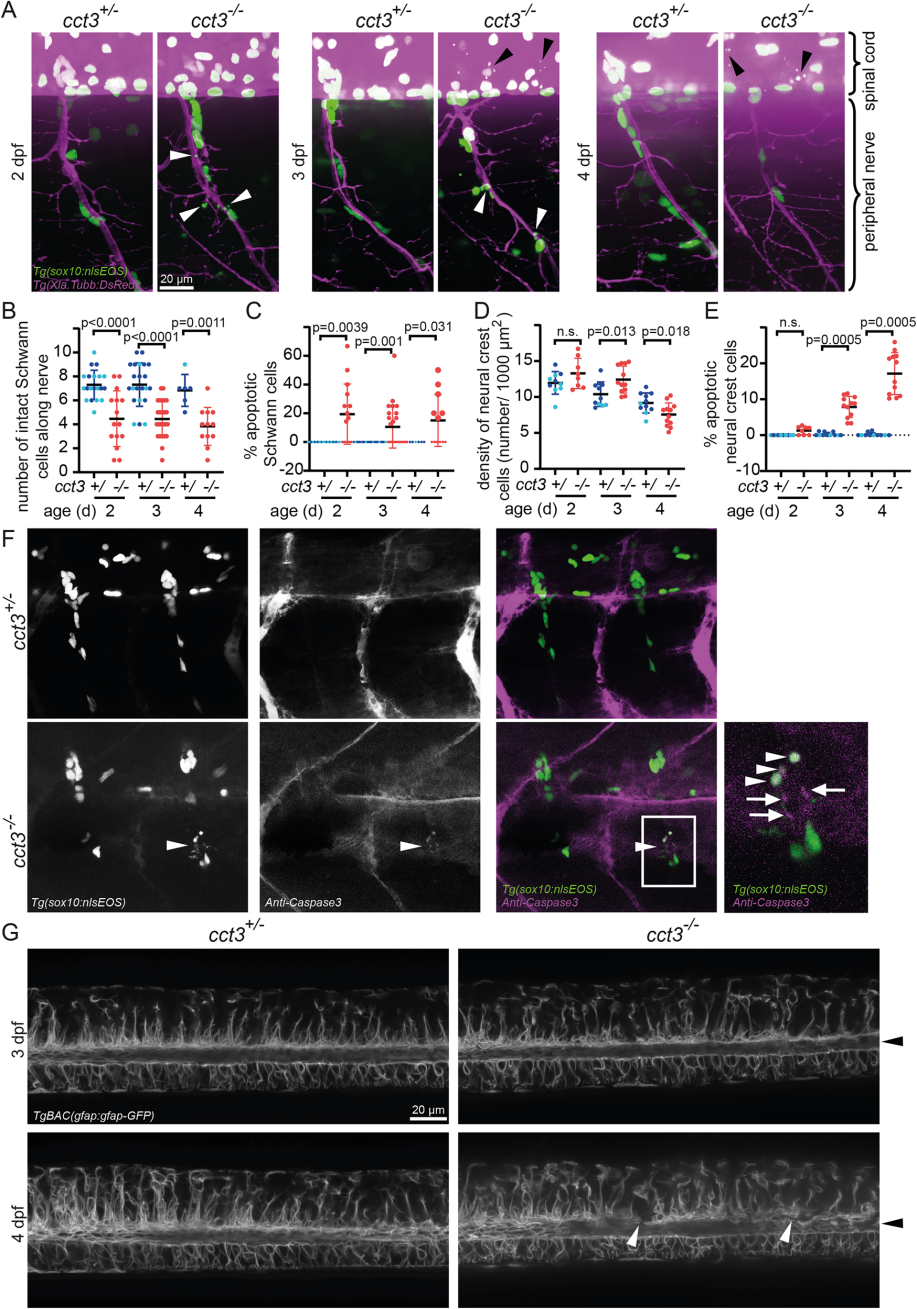
Cct3 is required for the survival of neural crest-derived cells. **A** Live imaging of double transgenic fish at 2, 3 and 4 dpf showing neurons and their axons in magenta *in tg(Xla.Tubb:DsRed)*^*zf148*^. No obvious morphological differences were observed between 2 and 4 dpf in *cct3* mutants compared to wt siblings. Nuclei of Schwann cells and their precursors are labeled by the *tg(sox10:nlsEOS)*^*w18Tg*^ transgene. Apoptotically fragmenting Schwann cell (and their precursor) nuclei are highlighted by white arrowheads, those of neural crest-derived cells that were still dorsal - at the level of the spinal cord - labeled by black arrowheads in *cct3* mutants. Quantitative analysis of the number of Schwann cells and their precursors along peripheral nerves (**B**), percentage of apoptotically fragmenting Schwann cell nuclei along peripheral nerves (**C**) density of neural crest cells at the level of the spinal cord (**D**), apoptotically fragmenting neural crest cell nuclei (**E**). Heterozygous were indistinguishable from wt (except for marginally significantly denser neural crest cells in heterozygous compared to wt, in contrast mutants displayed a reduction, *p* = 0.043, see Supplementary data file). Therefore siblings are displayed together - wt indicated by light blue data points and heterozygous individuals by dark blue data points. Unpaired Student’s *t* test (**B**, **D**, **E**) and Wilcoxon signed rank test (**C**) were used to determine *p* values. **F** To further demonstrate that fragmentation of nuclei of sox10^+^ cells is apoptotic cell death, we performed immunolabeling of activated Caspase 3 of *tg(sox10:nlsEOS)*^*w18Tg*^ fish at 3 dpf. A fragmenting EOS positive Schwann cell nucleus can be seen in a mutant larva, the same cell shows activated Caspase 3, partially, overlapping with the nuclear signal (white arrowheads), partially cytoplasmic (arrows), highlighted in the zoomed-in image on the right. This also shows that apoptotic cell death is not widespread in *cct3* mutants at 3 dpf. **G** GFAP-positive astroglial cells in the spinal cord of the transgenic *TgBAC(gfap:gfap-GFP)* zebrafish line showed focal irregularities (white arrowheads), especially along the central canal (black arrowhead) at 4 dpf in *cct3* mutants compared to siblings, but no overt abnormalities.

Based on the reduced white matter observed by MRI scans of patients with *CCT3* pathogenic variants, we aimed at understanding the role of *cct3* in myelin biology. In the transgenic line *Tg(mbp:EGFP-CAAX)*, membrane-attached green fluorescent EGFP is expressed in cells with an active *myelin basic protein (mbp)* promoter (Fig. 2A). These include myelinating cells in the central and peripheral nervous system. In wt siblings, we observed myelinating cells from 3 dpf (days post fertilization), first in the ventral and dorsal spinal cord. At 4 dpf, more myelinating cells appeared in the spinal cord, and the membrane segments, i.e. internodes were elongated. Furthermore, peripheral myelinating glia cells (myelinating Schwann cells) appeared around the ventrally projecting peripheral axons, where they displayed a typical segmental membrane shape (Fig. 2A). In the absence of functional *cct3*, there are considerably fewer myelinating cells in the ventral spinal cord, very few with the typical segmental/internodal pattern and very few and short processes extending into the ventrally projecting peripheral nerves (Fig. 2A). Electron microscopy confirmed normal myelin formation around central and peripheral axons in wt siblings in the spinal cord and in the lateral line (Fig. 2B). These results show that *cct3* is required for proper myelination in the central and peripheral nervous system in zebrafish. Since myelin forms around axons and axon-to-glia signaling is required for myelination, we sought to determine whether an obvious disruption of the axons would explain myelination impairment. For this, we focused on peripheral nerves, in which we can clearly visualize segmental ventrally projecting motor axon bundles in the *Tg(mnx1:GFP)* transgenic line and the membranes of surrounding neural crest-derived Schwann cells and their precursors in the *Tg(sox10:mRFP)* transgenic line (Fig. 2C). The outer appearance of peripheral motor neurons and their axons at 2, 3, and 4 dpf was normal in the absence of functional *cct3*. Likewise, the numbers and outer morphologies of motoneuronal cell bodies in the spinal cord were normal up to 4 dpf. In wt siblings, Schwann cells and their precursors displayed bipolar elongated cellular processes, accompanying these axons at 2 and 3 dpf. At 4 dpf, several Schwann cells shorten these processes and segmentally ensheath their associated axons, an internodal pattern of Schwann cells becomes discernible. In contrast, in *cct3* mutants, Schwann cells and their precursors frequently displayed a more rounded shape (arrowheads in Fig. 2C), shorter processes (brackets in Fig. 2C) and cellular fragmentation (white arrows along peripheral nerves in Fig. 2C) as early as 2 dpf, and more pronounced at 4 dpf, when they also fail to transform into the segmental/ internodal pattern. Likewise, in the spinal cord, cellular fragmentation of *Tg(sox10:mRFP)*^*+*^ cells becomes apparent from 3 dpf on (white arrows; spinal cord in Fig. 2C). This suggested that within peripheral nerves, Schwann cells are particularly susceptible to loss of *cct3*. Cellular rounding and shortening of processes suggested cytoskeletal changes, possibly apoptotic cell death [24]. Therefore, we further determined Schwann cell number and nuclear morphology in the *Tg(sox10:nlsEOS)* line (Fig. 3). The number of intact Schwann cell and precursor nuclei was significantly reduced at 2, 3 and 4 dpf in *cct3* mutants (Fig. 3A, B). This went along with a significant increase in the percentage of apoptotically fragmenting Schwann cell (precursor) nuclei (Fig. 3A, C). In addition to the Schwann cells associated with the peripheral nerves, the *Tg(sox10:nlsEOS)* line also labels neural crest cells that have not migrated that far yet, but that were still at the level of the spinal cord at 2, 3 or 4 dpf. Their numbers remained normal (at 2 dpf) or even slightly, possibly compensatorily, elevated (at 3 dpf) until 4 dpf, when they were finally also reduced in *cct3* mutants (Fig. 3A, D). From day 3, they also showed increased nuclear fragmentation (Fig. 3A, E). To demonstrate that Schwann cell nuclear fragmentation was indeed apoptosis and to determine how widespread apoptotic cell death would be beyond the nervous system, we performed whole-mount immunofluorescence to detect activated Caspase 3 in the *Tg(sox10:nlsEOS)* line. While activated Caspase 3 was undetectable in normally shaped Schwann cell nuclei, we detected activated Caspase 3 in apoptotically fragmenting Schwann cell nuclei at 3 dpf (Fig. 3F). In contrast to neural crest-derived cells, GFAP-positive astroglial cells in the spinal cord did not show any overt signs of degeneration or cell death at 3 and 4 dpf (Fig. 3G). Nevertheless, their processes extending to and aligning with the central canal (black arrowheads) occasionally appeared irregular (white arrowheads) in *cct3* mutants, especially at 4 dpf (Fig. 3G). Together, our data suggest that Schwann cell (precursors) along peripheral nerves and later also neural crest cells at the level of the spinal cord are particularly susceptible to loss of *cct3* function.

### Role of *cct3* in skeletal muscle and developmental refinement of neuromuscular junctions

**Fig. 4:**
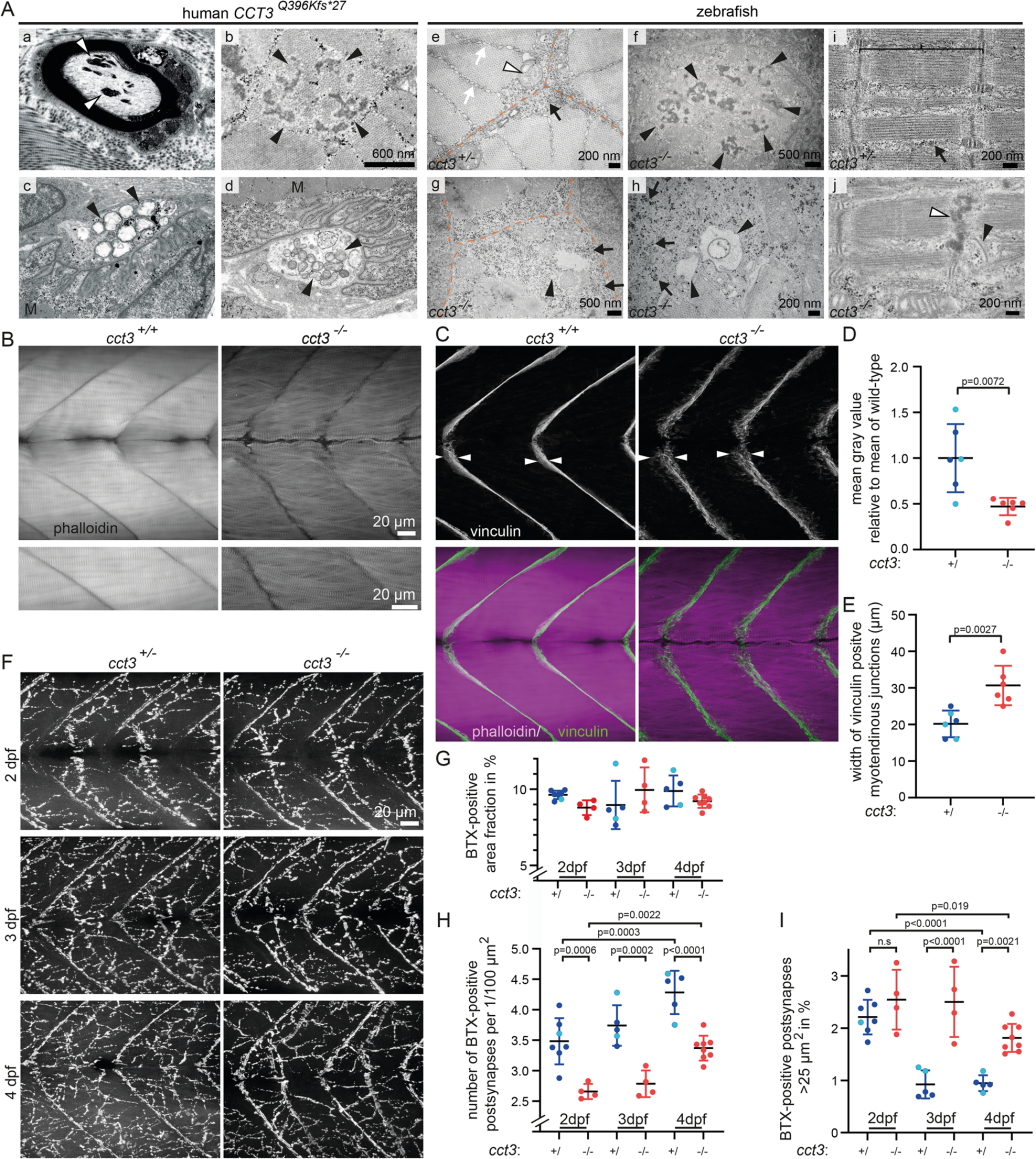
Role of cct3 in skeletal muscle and neuromuscular junction development. **A** Electron microscopy of skeletal muscle of a human patient with *CCT3* mutation (left, a-d) and of zebrafish (right, e-j). Normal structure of non-mutant sibling zebrafish skeletal muscle (e = cross section, i = longitudinal section), with normal sarcomere ultrastructure (curly bracket in i) and normal morphology of subsarcolemmal and intermyofibrillar cytoplasm with glycogen (arrow in e and i), mitochondria (white arrowhead in e), T-tubular system (white arrow in e) and other small vesicular organelles. Sarcolemma is labeled by interrupted orange line. In 4 dpf *cct3* mutants, EM shows enlarged deposits of sarcoplasmic glycogen as well as more and sometimes enlarged vesicles (glycogen labeled by black arrows and vesicles labeled by arrowheads; small ones in g and larger ones in h). Furthermore, EM showed focally abnormal sarcomere structure (black arrowhead in j) with abnormal buildup of disintegrated Z-band material in *cct3* mutants (between black arrowheads in f and white arrowhead in j), resembling the findings in the patient with *CCT3* mutation (b). Representative image of intraaxonal accumulations of abnormal autophagic material in a myelinated intramuscular nerve fiber in the patient´s biopsy (a). Motor end plates displayed a paucity of synaptic folds in and swelling of mitochondria and vesicular degeneration in axon endings (arrowheads in c-d) displaying. **B**, **C** Co-labeling of F-actin (phalloidin) and vinculin at 4 dpf. Quantification of phalloidin labeling intensity (**D**) and width of vinculin-positive myotendinous junctions (**E**). Unpaired Student’s *t* test was used for statistical testing in (**D**, **E**). Heterozygous animals were indistinguishable from wt and therefore siblings are displayed together—wt indicated by light blue data points and heterozygous individuals by dark blue data points. **F** Bungarotoxin (BTX)-labeling showing clusters of acetylcholine receptor at the postsynaptic side of the neuromuscular junction at 2 (upper row), 3 (middle row) and 4 dpf (lower row) in wt siblings (left column) and *cct3* mutants (right column). Quantification of the area fraction covered by BTX-positive synapses (**G**) density of BTX-positive postsynapses (number per area; **H**) and percentage of large BTX-positive postsynapses (>25 µm^2^) (**I**). ANOVA test followed by Šídák correction for multiple comparisons was used for statistical testing in (**G**–**I**). Heterozygotes were indistinguishable from wt and therefore siblings are displayed together—wt indicated by light blue data points and heterozygous animals by dark blue data points. Error bars show standard deviation. In siblings, over time, the density of synapses increases, while the area fraction of BTX-labeled postsynapses remains unchanged (no significant differences). This can be explained by a refinement of postsynapses during development, i.e. over time, more, but smaller BTX-positive acetylcholine clusters develop. In *cct3* mutants, the area fraction of BTX-labeled postsynapses is not significantly different from siblings. However, the density of synapses is significantly lower at all time points. This correlated with a higher percentage of larger BTX-positive acetylcholine clusters (>25 µm^2^), indicative of deficient refining of the postsynapses over time in *cct3* mutants.

We next correlated our previous observations of minor skeletal muscle, nerve fiber and neuromuscular junction abnormalities in a patient with the *CCT3* Q396Kfs*27 variant with those in zebrafish loss-of-function *cct3* mutants (Fig. 4). Electron microscopy showed normal skeletal muscle ultrastructure in cross sections in a wt zebrafish sibling. In zebrafish *cct3* mutants, next to sarcomers with mostly normal ultrastructure, we observed subsarcolemmal accumulation of glycogen- and vesicles. Focally altered sarcomere structure with buildup of disintegrated Z-band material was seen, resembling the findings in the patient with the *CCT3* Q396Kfs*27 variant. Given the role of Cct3/TRiC in folding of cytoskeletal proteins, we examined F-actin distribution in skeletal muscle by phalloidin staining, combined with immunohistochemistry for vinculin, an actin-binding protein that attaches muscle fibers at the myotendinous junction along the segment boundaries of zebrafish (Fig. 4B-E). In contrast to the F-actin irregularities that we had observed in hindbrains of *cct3* mutant zebrafish and in the intestine of *cct3* mutant *C. elegans* [1], obvious F-actin aggregates were not observed in mutant skeletal muscle. Skeletal muscle fibers harbor high amounts of F-actin compared to other tissues in both wt siblings and mutants. Nevertheless, staining intensities were significantly reduced in *cct3* mutant skeletal muscle, compared to wt siblings (Fig. 4B-D). Furthermore, myofibers containing arrays of sarcomeres between myotendinous junctions appeared more irregular in *cct3* mutants (Fig. 4B, C). Vinculin was more widely distributed and appeared frayed in *cct3* mutants compared to siblings, suggesting altered muscle fiber attachment at myotendinous junctions (Fig. 4B, E).

In the previously published patient-derived muscle biopsy, we had also observed accumulations of intraaxonal abnormal autophagic material, a paucity of synaptic folds, and swelling of mitochondria in axon endings (Fig. 4A [1]). To determine the consequences of TRiC malfunction on neuromuscular junction development over time, we performed Bungarotoxin (BTX) labeling of acetylcholine receptor clusters. In wt siblings, between 2 and 4 dpf, the density of synapses increases, while the area fraction of BTX-labeled postsynapses remained unchanged. This can be explained by a refinement of postsynapses during development. I.e. over time, larger BTX-positive acetylcholine clusters decline in siblings, while smaller, more refined and elaborated synaptic structures appear. In *cct3* mutants, the area fraction (area per area) of BTX-labeled postsynapses was not significantly different from siblings. However, the density (number per area) of synapses was significantly lower in *cct3* mutants at all time points examined. This correlated with a higher percentage of larger BTX-positive acetylcholine receptor clusters (>25 µm^2^), indicative of deficient refining of the postsynapses over time in *cct3* mutants (Fig. 4F–I) and in line with the observed paucity of synaptic folds in an affected patient [1].

### *Cct3* is required for axonal cytoskeleton integrity and axonal transport

**Fig. 5:**
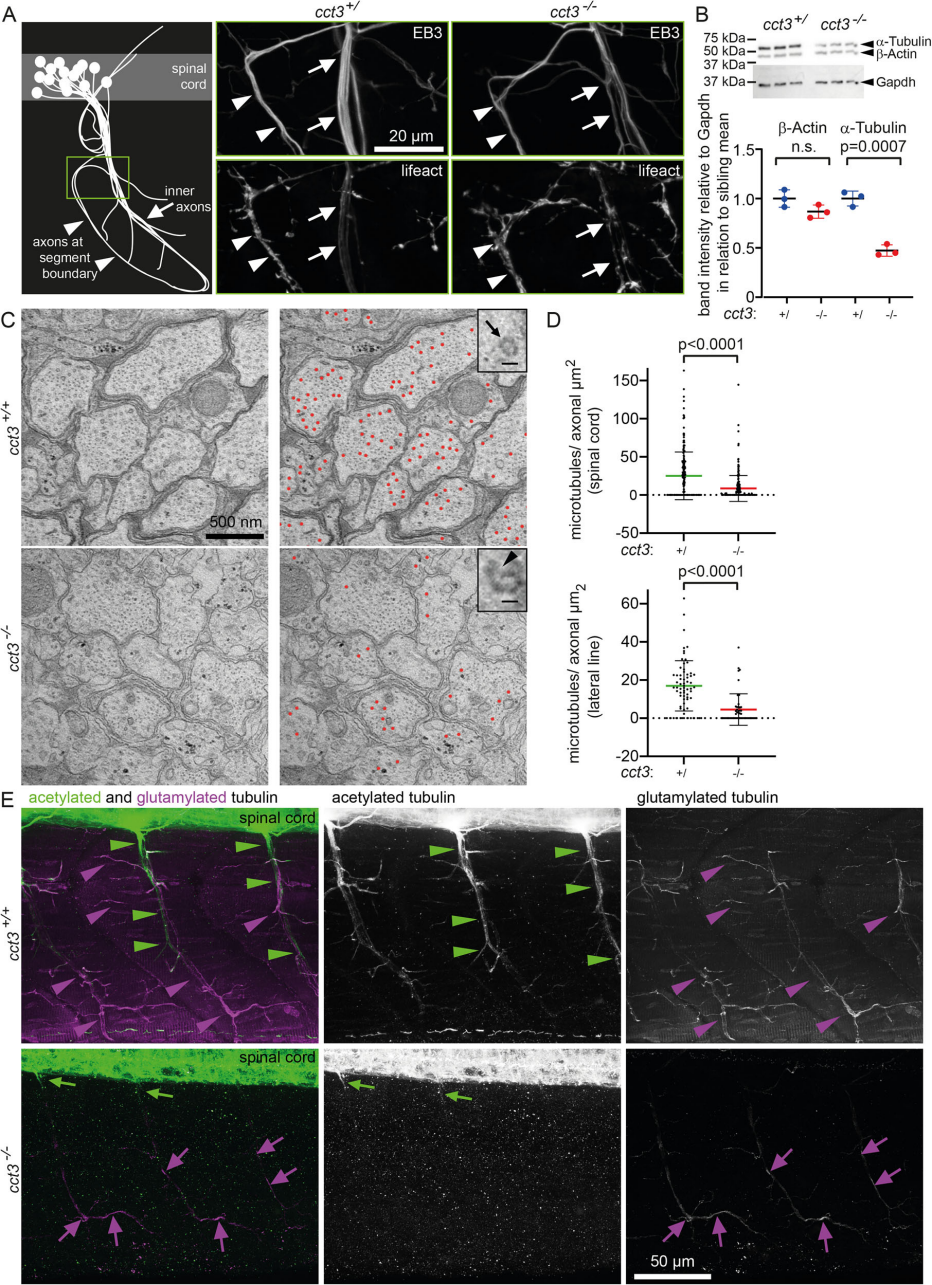
Cct3 is required for axonal F-actin and microtubules. **A**-**D** F-actin and microtubules are visualized in double transgenic zebrafish: *Tg(mnx1:Gal4)*; *Tg(UAS:lifeact-GFP-p2a-EB3-RFP)*^*p412Tg*^. **A** Scheme showing the motoneuronal cell bodies in the spinal cord with their axons extending into the peripheral nerves at the inner side of the myotome, axon “trunk” (inner axons, arrow) and also between the segment boundaries (arrowheads). Region of interest shown in B is marked by a green box within the schema in (**A**). **B** Strong EB3 labeling in neurons along axonal microtubules in siblings and *cct3* mutants without any obvious structural defects at this magnification and location. While F-actin appears irregular along the segment boundaries in both, siblings and *cct3* mutants (arrowheads), the inner axons have more regular F-actin distribution in siblings, but irregularities in *cct3* mutants. (**B**, lower row, quantification provided in Supplementary Fig. 2) **B** Western blot (same membrane as in Fig. 1E) was incubated with anti-β-Actin and anti-α-Tubulin. Compared to Gapdh, both were reduced, with β-Actin showing a slight, non-significant trend to reduction, but α-Tubulin being reduced to ~50% of wt levels. For full membranes, see Supplementary Original data. **C**, **D** Electron microscopy of the spinal cord and lateral lines axons revealed strongly reduced normally shaped microtubules in *cct3* mutants compared to wt larvae at 4 dpf. Exemplary representative images of the spinal cord (zoomed-in images of those previously shown in Fig. 2B) are shown without (left column) and with marked microtubules of the expected shape and size around 25 nm in diameter (red dots; right column). Furthermore, we show a single microtubule in maximal magnification in the upper right corner—normal morphology (arrow in wt) and abnormal tubular structure with interrupted configuration frequently observed in mutants (arrowhead), presumably representing abnormal microtubules; scale bar = 25 nm. **D** Number of microtubules per axonal area (µm^2^) in spinal cord axons and the axons of the lateral line in mutants and siblings (*n* = 3 each, Mann-Whitney test was used to determine *p*-values). Heterozygotes were indistinguishable from wt and therefore siblings are displayed together (see Supplementary data file). **E** Co-immunolabeling of acetylated and polyglutamylated tubulin of siblings (*n* = 6) and mutants (*n* = 8).

Given the role of TRiC in the folding of actin and tubulin on the one hand and the functional significance of these cytoskeletal components in neurons on the other hand, we next assessed F-actin and microtubules in motor axons of peripheral nerves (Fig. 5). First, we generated a double transgenic line, combining motor neuronal Gal4 expression [*Tg(mnx1:Gal4)*] with the previously established *Tg*(*UAS:lifeact-GFP-p2a-EB3-RFP)*^*p412Tg*^ line and analyzed cell bodies along with axon “trunks” inside the myotome (“inner axons”) and those closer to the surface, along the segment boundaries, respectively (Fig. 5A). In these fish, we determined localization and distribution of the microtubule plus end-binding protein EB3 and of F-actin. EB3 continuously labeled microtubules along the axons—a pattern known upon its overexpression [25]—in both, wt siblings and mutants, with a non-significant trend towards weaker signal in *cct3* mutant axons (Fig. 5A, upper row). F-actin patterns in axons along the segment boundaries were irregular in both, siblings and mutants, possibly reflecting ongoing remodeling at these synaptic locations (arrowheads in Fig. 5A, lower row). In contrast, the “inner axons”, inside the myotome, showed an even distribution of F-actin in wt siblings, while *cct3* mutants displayed an irregular pattern at this location as well (arrows in Fig. 5B, lower row, Supplementary Fig. 2). This suggests an altered F-actin architecture in *cct3* mutants in line with the previously observed F-actin irregularities in *C. elegans* intestine and zebrafish hindbrain [1]. These alterations prompted us to examine actin and tubulin as well as microtubules in more detail. Western blot analysis revealed a non-significant reduction of β-actin, but a strong and significant reduction of α-tubulin in *cct3* mutants compared to siblings (Fig. 5B). Next, we quantified microtubules in the ultrastructural images. Normal microtubules were determined based on their tube-like morphology in cross sections with a typical diameter of around 25 nm. In the absence of functional *cct3*, we observed a significant and strong reduction of normally shaped microtubules in peripheral and central axons (Fig. 5C, D). Since posttranslational modifications of tubulin are crucial for the function of microtubules, we determined acetylated and polyglutamylated tubulin by whole-mount immunolabelling (Fig. 5E). In wt siblings, acetylated tubulin was highly abundant in the spinal cord. In the peripheral nerves, acetylated tubulin was mainly detectable in the more central nerve trunk (“inner axon”) and in the proximal regions of its branches (green arrowheads). In contrast, in the wt siblings, the polyglutamylated tubulin was enriched in the more distal regions of the axonal branches (magenta arrowheads), but not at the segment boundaries either, while less polyglutamylated tubulin was present in the “inner axon”, giving both, acetylated and polyglutamylated tubulin an inverse appearance in the peripheral nervous system in wt siblings at 4 dpf. While acetylated tubulin was detectable in *cct3* mutant spinal cords, there was very little immunoreactivity just adjacent to the motor exit points (green arrows), but not along the “inner axons” in the peripheral nervous system of *cct3* mutants. Compared to wt siblings, polyglutamylated tubulin showed a weaker immunolabelling, but it was present (in contrast to acetylated tubulin) in the mutants—with an accentuation also in the more distal regions of the “inner axons” (magenta arrows).

Together, these data show that despite apparently normal outer motor axonal morphology in peripheral nerves, the cytoskeleton shows severe alterations in *cct3* mutants and in particular a severe reduction of acetylated tubulin.

**Fig. 6:**
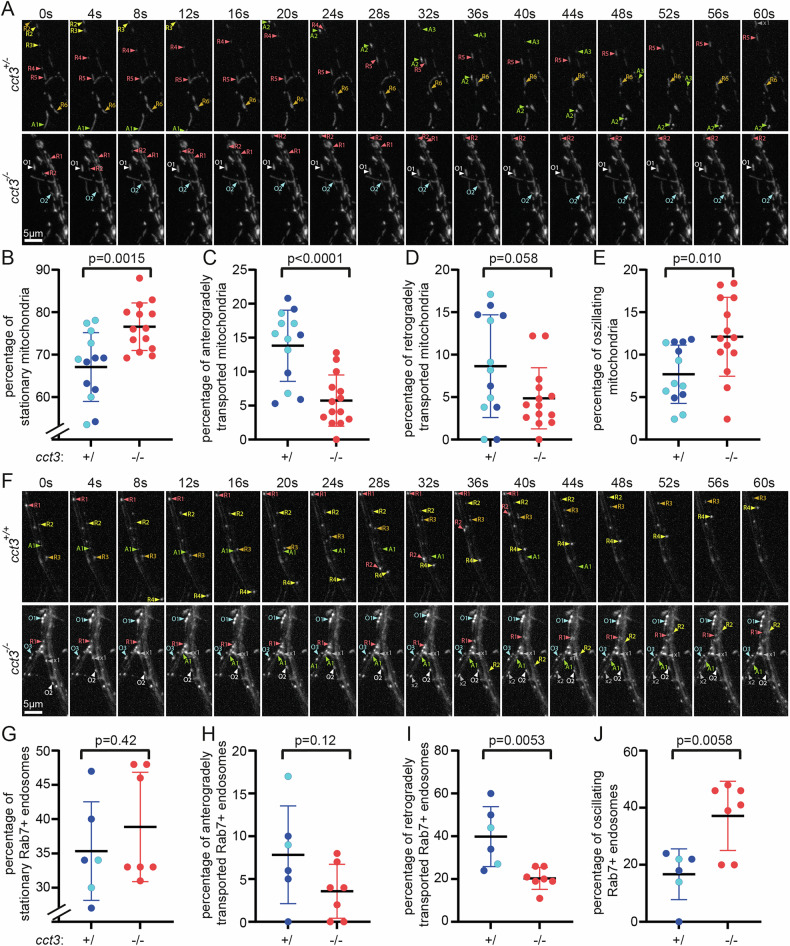
Cct3 is required for axonal organelle transport. Motoneuronal, i.e. axonal mitochondria and Rab7+ endosomes were labeled by the combination of *Tg(mnx1:Gal4)*, expressing Gal4 in motoneurons with either *Tg(UAS:mito-mCherry)* to label mitochondria (**A**–**E**) or *Tg(UAS:EGFP-Rab7)* to label Rab7+ endosomes (**F**–**J**). Small regions of the time lapse images of representative sibling and *cct3* mutant axons are shown in (**A**) (mitochondria) and F (endosomes) over time, at 4 s intervals. Discernible moving organelles are highlighted by yellow, orange and red arrowheads for retrogradely transported organelles (numbered R1-6), green arrowheads for anterogradely transported organelles (A1-3), white or blue arrowheads for oscillating organelles (O1-3) and gray arrowheads for movement with undefined characteristics, e.g. short movement followed by stalling (x1-2). Full movies of three examples per organelle and genotype are shown in the Supplementary movies 1 and 2. Quantifications of stationary organelles, retro- and anterogradely transported organelles as well as oscillatory movements are shown below the images, in (**B**-**E**) for mitochondria and in (**G**-J) for the endosomes. Heterozygous animals were indistinguishable from wt and therefore siblings are displayed together—wt indicated by light blue data points and heterozygous individuals by dark blue data points. Unpaired Student’s t-test was used as statistical test, mean and standard deviation are shown.

Given the essential role of *cct3* for axonal microtubule density and tubulin acetylation, both of which have a known essential role in axonal transport, we hypothesized that *cct3* would be required for axonal transport. We generated two transgenic zebrafish lines, *Tg(UAS:mito-mCherry)* and *Tg(UAS:EGFP-Rab7)*, which in combination with the *Tg(mnx1:Gal4)* line label motoneuronal mitochondria and endosomes, respectively (Fig. 6, Supplementary movie 1 and 2). We determined axonal transport by performing time-lapse imaging at 3 dpf. Axonal transport of both mitochondria (Fig. 6A-E) and Rab7+ endosomes (Fig. 6F-J) was significantly reduced in *cct3* mutants compared to wt siblings. In contrast to the fast and directed transport in wt siblings, *cct3* mutants frequently showed oscillating movement of organelles, in line with disturbed transport routes, i.e. disruption of microtubules.

## Discussion

Patients with TRiCopathies, especially those caused by pathogenic variants in *CCT3*, display reduced white matter in brain MRI scans [1]. Similarly, we showed that in zebrafish, loss of Cct3 function was associated with a profound reduction of myelin formation and of myelinating cells in both the central and peripheral nervous system. This reduction in myelinating cells was associated with severe morphological abnormalities and apoptotic nuclear fragmentation of sox10^+^ neural crest-derived Schwann cell precursors and Schwann cells along peripheral nerves as well as oligodendrocyte precursor cells in the spinal cord. In contrast, spinal motor neurons and astroglial cells did not show any overt signs of cell death until at least 4 dpf. Therefore, sox10^+^ cells, including myelinating cells, are particularly vulnerable to loss of Cct3 function, suggesting that their decreased survival contributes to white matter pathology in patients with TRiCopathies in general and those with *CCT3* mutation in particular.

TRiCopathies described by Kraft et al. [1] show dominant inheritance, while we observed a loss-of-function phenotype in our zebrafish *cct3* mutants. This is likely explained by either haploinsufficiency or a dominant negative effect of the identified human mutations, while heterozygous zebrafish larvae were indistinguishable from wt in our analyses. However, recessive mutations in *CCT5* were previously shown to be linked to autosomal recessive mutilating sensory neuropathy with spastic paraplegia and spinal cord atrophy [26], demonstrating that recessive inheritance/loss-of-function is also relevant in patients with *CCT* mutations. Electrophysiological examinations revealed a predominantly axonal neuropathy with normal to slightly reduced nerve conduction velocities in these patients [27]. In fact, we also observed axonal alterations that were more subtle than the lethal phenotype of *cct3*-deficient myelinating glial cells, primarily affecting the axonal cytoskeleton, i.e. F-actin and microtubules. Furthermore, the essential role of *cct3* for microtubule density, tubulin protein amounts and posttranslational modification (in particular its acetylation, which in turn regulates many cellular functions, including axonal transport [28]), explains at least in part the neurodevelopmental disturbances in patients with TRiCopathies. In line with this concept, patients with dyneinopathies, carrying mutations in the *DYNC1H1* gene that is essential for retrograde (axonal) transport, display a similarly broad spectrum of neurological and neurodevelopmental disorders as patients with TRiCopathies [1, 29]. In fact, dyneinopathies and TRiCopathies share a majority of phenotypes such as abnormal cortical gyration/polymicrogyria, white matter changes, corpus callosum dysgenesis, cerebellar hypoplasia and axonal neuropathy [1, 29].

We have previously identified a more subtle effect on neuromuscular junction formation in patients with a pathogenic *CCT3* variant [1], showing a paucity of synaptic folds. Here, we showed that this is evolutionarily conserved, since we observed similar defects in *cct3* loss-of-function zebrafish embryos and larvae. I.e., we found that *cct3* is required for synaptic refinement in the developing neuromuscular junction. A central player in this synaptic refinement is agrin, a protein that is released from the presynaptic nerve terminals and binds to its postsynaptic low-density lipoprotein receptor LRP4, which in turn stimulates the kinase MuSK. Agrin is transported from the cell body along axons either as protein or as mRNA for presysnaptic translation, requiring intact axonal transport [30, 31]. A synthetic agrin fragment was shown to rescue a similar synaptic phenotype (fewer, but larger AChR clusters) in zebrafish deficient in myosin 9a, which is hypothesized to control cytoskeleton, i.e. transport and actin-crosslinking in neurons [32]. Hence, altered axonal transport of agrin could contribute to the synaptic alterations in TRiCopathies.

In line with previous observations in zebrafish mutants of different TRiC subunits [33], we found that the F-actin signal was reduced in skeletal muscle of *cct3* mutants. However, although F-actin was highly abundant in skeletal muscle, we did not observe focal irregularities or enrichments within skeletal muscle fibers (Fig. 4) like those observed in *cct3*-mutant *C. elegans* intestine and zebrafish hindbrain [1] or motor axons (Fig. 5). Likewise, no alpha-actin-GFP aggregates were observed in skeletal muscle cells upon transgenic overexpression in a previous study of *cct* mutant zebrafish. In apparent contrast, in the same study, TRiC has been shown to be required for alpha-actin folding, i.e. TRiC was essential for nemaline rod formation when nemaline myopathy-associated mutant *ACTA1* was expressed [33]. The most plausible explanation for this apparent contrast, i.e. the absence of F-actin focal irregularities in skeletal muscle despite the role of TRiC in alpha-actin folding, is that misfolded alpha-actin is more quickly degraded and removed in skeletal muscle fibers than other actin isoforms in other tissues [33].

We examined the morphology of different neural cell types in the spinal cord and peripheral nerves, including neurons, astroglial cells and neural crest-derived cells, including Schwann cells. At day 2, we observed apoptotic nuclear fragmentation specifically of those neural crest-derived cells that had exited the spinal cord and migrated along peripherally projecting axons, i.e. Schwann cells and their precursors. From day 3 onward, we also observed apoptosis of neural crest cells at the level of the spinal cord, while neurons and astroglial cells remained virtually intact up to 4 dpf. This suggests a particular susceptibility of Schwann cells and their precursors to *cct3*-deficiency. The reason for this selective vulnerability remains elusive, but it is tempting to speculate that there is a causal link between increased cell death and the particularly high migratory activity of these cells while leaving the neural crest and growing along peripheral nerves that requires highly dynamic cytoskeletal remodeling, which depends on TRiC for proper protein folding of cytoskeletal proteins. Alternatively, axonal transport alterations due to *cct3* mutations might alter axonal distribution of gliotrophic factors such as neuregulin 1. Neuregulin 1 is crucial for axon-glia signaling and required for Schwann cell survival and myelination, and even neuromuscular junction formation [34].

In summary, our findings significantly enhance our understanding of the conserved role of Cct3/TRiC in the developing nervous system and the pathophysiological mechanisms in TRiCopathies. We hypothesize that the essential role of Cct3 in myelination, survival of neural crest-derived cells and NMJ refinement could have a common denominator, namely the essential role of Cct3 in axonal transport that we demonstrated. Future studies will show whether components involved in axon-glia signaling and those mediating NMJ refinement are aberrantly transported and localized in *cct3* mutant axons and whether aberrant transport and location are causing the other observed phenotypes.

## Materials and methods

### Ethics statement

All zebrafish experiments complied with the guidelines of the State of North Rhine-Westphalia (Germany), the Bundesinstitut für Risikobewertung and were approved by the Landesamt für Verbraucherschutz und Ernährung (LAVE) under permits numbers 81-02.04.2021.A304, 81-02.05.40.21.001, and 81-02.04.40.2023.VG090.

The study (use of patient-derived material) was approved by the institutional ethical review boards at the medical faculty of Aachen under the permit number EK302-16. The research included in this report was performed in a manner consistent with the Declaration of Helsinki and/or the Belmont Report. The legal guardians provided informed consent to participate in the study.

### Zebrafish care and strains

Embryos were generated by natural mating as described [35]. All embryos were raised at 28 °C. The *Tg(mnx1:GFP)*^*ml2*^ [36] was used to label peripheral motor nerves. Alternatively, peripheral nerves were visualized using the Tg(*Xla.Tubb:DsRed*)^*zf148*^ [37] transgene with pan-neuronal dsRed expression. Neural crest-derived cells, including Schwann cells were labeled using the *Tg(sox10:mRFP)*^*vu234*^ [38] (RFP-labeling of membranes), and *Tg(sox10:nlsEOS)*^*w18Tg*^ [39] (nuclear labeling with EOS). Myelinating cells with activated MBP promoter were visualized in the *Tg(mbp:EGFP-CAAX)*^*ue2*^ line [40], and GFAP-positive astroglial cells were labeled in the *TgBAC(gfap:gfap-GFP)*^*zf167*^ line [41].

To drive Gal4 expression in motor neurons, we generated Tg(*mnx1:Gal4*) zebrafish lines, using I-SceI (meganuclease). This construct contained two copies of *mnx1:Gal4* since this was found to increase expression profoundly [42]. This transgene was combined with the previously generated line *tg(UAS:lifeact-GFP-v2A-EB3-RFP)*^*p412*^ [43].

For visualization of mitochondria and endosomes, we generated two new fish lines, *Tg*(*UAS:mito-mCherry*) and *Tg*(*UAS: EGFP-rab7a*). For this, we amplified zebrafish *rab7a* cDNA and cloned this into the pEntr plasmid. We used an existing pEntr containing mito-mCherry. To transfer these elements into Destination vectors containing the UAS promoter, a destination cassette followed by the SV40 poly A as well as the *cmlc2:EGFP* construct, we used Gateway cloning, i.e. LR clonase II (Invitrogen) [44]. The resulting plasmids were microinjected into embryos at the one cell stage together with Tol2 RNA for generation of transgenic founders [45]. Both lines were combined with *Tg(mnx1:Gal4)* in order to label mitochondria and Rab7^+^ endosomes in mnx1-expressing motor neurons, respectively.

We generated loss-of-function alleles of *cct3* using CIRSPR/Cas9 with the target sequence: 5’-GGTCATGACGATACCACCCATGG-3’ located in exon 4 of the cct3 gene as previously described [1, 46]. Single guide RNAs were cloned using the DR274 plasmid. The established zebrafish line was further characterized by sequencing, revealing a 4 bp deletion in exon 4 (NM173250.1:c.161_164del), causing a frameshift mutation. This mutation resulted in the substitution of glycine (G) with valine (V) at position 54, leading to a premature stop codon at position 57 (cct3p.G54Vfs*4). Additionally, a second zebrafish line harboring a 4 bp deletion in exon 4 (NM173250.1:c.163_166del) leading to a frameshift mutation was generated. In this line, the mutation caused a substitution of glycine (G) with serine (S) in position 55, resulting in a premature stop codon at position 57 (cct3p.G55Sfs*3).

### Genotyping

We extracted DNA from fin biopsy material in 100 µl and from whole larval tissue in 20 µl of lysis buffer, pH 8.3 (150 mM MgCl_2_, 1 M Tris pH 8.3, 1 M KCl, 0.1% Gelatin, 0.3% Tween20, 0.3% NP40 and 100 µg/ml Proteinase K) by incubating them at 55 °C for 6 to 18 h. We inactivated the proteinase K afterwards at 95 °C for 10 min. We used GoTaq® (Promega) Taq polymerase for DNA amplification using the following dCAPS primers and the respective restriction enzymes for digest. For the distinction between wild-type from both mutant alleles (cct3p.G54Vfs*4 and cct3p.G55Sfs*3), forward primer CAGATGCTGCTGGATCCGACGG, reverse primer GGATGCTGGACCTGGATCTAAA, and DrdI restriction enzyme (New England Biolabs) were used. For distinguishing specifically the cct3p.G55Sfs*3 allele from the other two (wildtype and cct3p.G54Vfs*4) we used the dCAPS primers GATGCTGCTGGATCGAATGGGT (forward) and GGATGCTGGACCTGGATCTAAAGT (reverse) along with the XmnI restriction enzyme (New England Biolabs). Both PCRs were conducted with 35 cycles and an annealing temperature of 54°C in case of cct3p.G54Vfs*4 and 58°C for line cct3p.G55Sfs*3 (94 °C for 30 s, 54 °C/58 °C for 30 s, 72 °C for 30 s).

### *Cct3* mRNA synthesis and microinjection

*Cct3* cDNA was amplified from zebrafish cDNA, using the following primers: *cct3* fw: 5’-GCC ACC ATG ATG GGC CGA CCG GTT CTC-3’ and *cct3* rev: 5’-CTA CTC TCT GTC CTC CAT CGG TG-3’ and TA-TOPO cloned into pCR2.1. CDNA was then transferred into the pCS2+ plasmid, using EcoRI cutting sites. After linearization of pCS2 + -*cct3* with HpaI, cct3 mRNA was synthesized in vitro using the mMESSAGE mMACHINE™ SP6 Transcription Kit [Thermo Fisher Scientific, #AM1340] following the manufacturer’s instructions. The synthesized mRNA was purified by lithium chloride precipitation. Aliquots were stored at −80 °C until use. For microinjection, one-cell stage zebrafish embryos were collected. One nanoliter volume containing 300 pg cct3 mRNA, along with 0.1 M KCl and phenol red, was injected into the yolk of one-cell stage embryos using the FemtoJet 4x microinjector (Eppendorf). Uninjected embryos from the same clutches were used as controls. Injected versus non-injected embryos were randomly assigned to the groups.

### Live imaging and time-lapse microscopy

Live embryos and larvae were anesthetized in 0.006% tricaine, mounted in 1.0–2% agarose in E3 into Nunc™ glass bottom dishes (12 mm), and kept at 28 °C. Live larvae were imaged using a spinning-disk confocal microscope (Nikon) using VisiView software (Visitron).

For time-lapse microscopy of the double transgenic line *Tg(mnx1:Gal4) Tg(UAS:rab7-EGFP)*, images were acquired at 37 time points with 4.9-s interval over a total duration of 3 min, using a 49-step z-stack with a step size of 1 µm. Similarly, imaging of the double transgenic line *Tg(mnx1:Gal4) Tg(UAS:mito-mCherry)* was performed at 16 time points with a 4.0-s interval over a total duration of 1 min, using a 20-step z-stack with a step size of 1 µm.

### Electron microscopy

Zebrafish larvae were fixed overnight in 4% paraformaldehyde and 2% glutaraldehyde in 0.1 M sodium cacodylate buffer. After rinsing in 0.1 M phosphate/0.1 M sodium cacodylate buffer for 5 min., samples were incubated in 1% osmium tetroxide in 0.15 M phosphate buffer for 1 h. After rinsing twice in 0.1 M phosphate/0.1 M cacodylate buffer for 5 min. each, samples were dehydrated in increasing concentrations of ethanol (25%, 35%, 50%, 70%, 85%, 95%, absolute), each concentration was applied twice, 20 min. each. This was followed by a brief incubation in propylenoxide and then a 20 min incubation in 50% propylenoxide/50% epon mixture, with epon mixture being composed of 24 ml glycidic ether, 13.5 ml dodecenylsuccinic acid anhydride, 12.5 ml methylnadic anhydride and 0.8 ml 2,4,6-tris(dimethylaminomethyl)phenol and mixed for at least 1 h and until the solution turns amber colored. Afterwards, the larvae were incubated for 20 min in the epon mixture and then transferred into epon blocks, which were polymerized in an incubator for 9 h at 28 °C, 2 h at 80 °C, 4 h at 22 °C and lastly 2 days at 100 °C. Semithin sections (800 nm) from the larvae tissue were cut with the “Reichert Ultracut S” ultramicrotome by Leica (Wetzler, Germany). Next, each section was stained for 45–60 s at 80 °C with a methylene blue-azure II stain. After examination of these semithin sections using light microscopy, ultrathin sections (50 nm) were prepared. For that, the sections were rinsed in distilled water and then contrasted for 10 min with 0.5% uranyl acetate by Electron Microscopy Sciences (#22400) before washing three times in distilled water. Then, the sections were contrasted with lead citrate (Sigma-Aldrich, #228621) for 1 min and washed three times again. We also examined ultrathin sections of a human glutaraldehyde-fixed, epoxy resin-embedded muscle biopsy from a patient with the *CCT3*^Q396Kfs*27^ mutation. Ultrastructural examination was performed using a Hitachi HT7800 transmission electron microscope.

### Immunohistochemistry

For visualization of F-actin in the skeletal muscle and vinculin at the myotendinous junction, as well as acetylated and glutamylated tubulin, zebrafish larvae were fixed for 2 h at RT in 4% PFA PBS-DT^1^ (1X PBS, pH 7.4, 1%DMSO, 0.2% Triton X-100) and washed in PBST^1^ (1X PBS, 0.2% Triton X-100). Depending on their developmental stage, larvae were permeabilized on ice with 1 mg/ml collagenase in 0.1 M phosphate buffer for 45 min (2 dpf), 60 min (3 dpf), and 75 min (4 dpf). Following permeabilization, larvae were washed again several times in PBST^1^ and incubated with blocking solution (5% goat serum in PBS-DT) for 2 h at RT. For vinculin/ actin co-labeling, larvae were incubated overnight at 4 °C with an anti-vinculin primary antibody (clone VIIF9 (7F9), Merck Millipore, MAB3574) at a 1:400 dilution in blocking solution. After four washed with PBST^1^, larvae were incubated overnight at 4 °C with TRITC-conjugated phalloidin (Merck Millipore, 90228) diluted in blocking solution at 1:1000. For acetylated/glutamylated tubulin staining, After subsequent washes in PBST^1^, the larvae were mounted in Vectashield® mounting medium (Vector Laboratories, H-1000-10) on glass slides and stored at 4 °C.

Acetylcholine receptor (AChR) clusters at the neuromuscular junction (postsynapse) were labeled with α-bungarotoxin (α-BTX) [47]. Not more than ten larvae together were fixed 4% PFA PBS-DT^2^ (1X PBS, pH 7.4, 1%DMSO, 0.1% Tween20) for 3 h at RT and subsequently washed several times in PBST^2^ (1X PBS, 0.1% Tween20). Depending on their developmental stage, larvae were permeabilized on ice with 1 mg/ml collagenase (Sigma-Aldrich; #C9891) in 0.1 M phosphate buffer for 45 min (2 dpf), 60 min (3 dpf), and 75 min (4 dpf). Afterwards, the larvae were incubated in pre-absorbed α-BTX Alexa Fluor^TM^ 488 conjugate (1:100; Invitrogen/Thermo Fisher Scientific; #B13422) in blocking solution at 4 °C ON. Pre-absorbed α-BTX was obtained by incubating wild-type (wt) larvae with α-BTX at 4 °C ON prior to use. The next day, the larvae were washed four times for 5 min in PBST^2^ and mounted in Vectashield mounting medium (Vector Laboratories, H-1000-10) on a glass.

### Image processing and data analysis

Confocal images were first processed using the ImageJ software package (NIH). Images were exported and further processed in Photoshop CS4. Image manipulations included generation of maximum intensity Z projections as well as adjustment of brightness and contrast, enhancement of contrast (processing in ImageJ/FIJI), and gamma-value (ImageJ/FIJI). Manipulations were always applied to the entire image and to all images in one experiment, ensuring that the content of the image was not altered. Final versions of the figures for the manuscript were prepared using Illustrator CS4 and Photoshop CS4 (Adobe).

### Quantification of lifeact signal in Mnx1^+^ motoneurons

A region of the inner nerve stump just below the horizontal myoseptum was selected and processed in FIJI to find maxima with a prominence of >50.00. Maxima within tolerance were counted and are displayed in Supplementary Fig. 2.

### Quantification of microtubules in central and peripheral axons

The quantification of axonal microtubules in ultrastructural images of the spinal cord and the lateral line was performed using ImageJ. Individual axons in EM images were marked up using the “polygon selection” tool and the surface area was measured using the “measure” function. In each of the labeled axons, identified microtubules were counted manually utilizing the ImageJ “cell-counter” plugin.

### Western blot

At 4 dpf, 10 siblings and 10 *cct3*-mutants were collected per tube in sample buffer (4 M Urea, 10% Glycerol, 4% SDS, 0.125 M Tris-HCl (pH 6.4), 100 mg bromphenolblue, 10% β-mercaptoethanol) containing protease inhibitor (Sigma, P8340). Samples were denatured by heating at 60 °C for 10 min, followed by immediate cooling on ice. After centrifugation (13,000 rpm, 5 min, 4 °C), supernatants were collected. Fifteen µl of the protein lysate was loaded per sample and separated on 4% and 10% SDS-PAGE. Proteins were transferred onto a PVDF membrane by wet blotting in transferring buffer (10%TBST, 20% MeOH). Membranes were incubated at room temperature (RT) for 15 min in Ponceau S. After washing with H_2_O, membranes were incubated for 1 h at RT in blocking buffer (1% (w/v) Blocking Reagent (Roche, 1096176), 20 mM maleic acid, 30 mM NaCl, 0.8 × TBST, 0.01% thimerosal). Incubation with primary antibodies was performed over night at 4 °C (TCP-1 γ antibody [1:1000; Santa Cruz; sc-271336], anti-beta actin [1:10,000; Abcam, ab6276], anti-α-Tubulin [1:1000; Sigma, T5168], anti-GAPDH [1:10,000, Abcam, EPR16891]). After washing 1× TBST membranes were incubated at RT for 2 h with HRP-conjugated secondary antibodies (goat anti-mouse IgG [1:2000; Invitrogen; #32430], Goat anti-Rabbit IgG [1:1000; Invitrogen; #31460]). After washing in 1xTBST, band detection was performed using the SuperSignal™ West Femto Maximum Sensitivity Substrate (Thermo Scientific™, #34095) and developed using the iBright™ FL1500 Imaging System (Invitrogen™).

### Quantification of organelle transport in Mnx1^+^ motoneurons

Motoneuronal transport was quantified in transgenic zebrafish lines labeling axonal mitochondria (*Tg(UAS:mito-mCherry))* and Rab7+ endosomes (*Tg(UAS:EGFP-Rab7)*) using ImageJ. The analysis was performed on time-lapse images acquired at 4-s intervals in the region of the inner axon trunc. Organelles were manually labeled in t-stacks consisting of 16 frames for mitochondria and 20 frames for Rab7+ endosomes using the “cell counter” plug-in. The transport behavior of each individual organelle was categorized according to five categories. First, stationary organelles remained fixed in place within the axon. Second, anterogradely transported organelles moved from the soma towards the axon terminals. Third, retrogradely transported organelles moving in the opposite direction. Fourth, organelles exhibiting oscillatory movement, characterized by a back-and-forth motion over a short range without evident direction. Fifth, organelles displaying long-distance movement with a directional change throughout the time-lapse. Organelles categorized into group five were excluded from further quantitative analysis since clearly recognizable movement fitting this behavior was only sporadic in both siblings and mutants. However, these organelles were included into the total number of analyzed organelles for each position.

### Sample size and statistical analysis

Since the effect size was not known prior to this study, power could not be determined a priori. We always analyzed a minimum of three individuals per group. Numbers are provided in the graphs (data points), in the figure legends and in the supplementary data file. For the organelle transport, only those images could be analyzed that had a good signal intensity, did not move throughout the timelapse and in which axons were clearly discernible. Otherwise no samples were excluded from the analysis. Blinding was not possible, because the mutant group was clearly visible to the researcher based on the phenotype.

*P*-values were calculated by the Fisher’s exact test for categorical data using a GraphPad web tool (GraphPad), by the Student’s *t* test, the Mann-Whitney test or by the ANOVA test using GraphPad software (GraphPad) as indicated in the figure legends. Graphs were generated using Prism 10 (GraphPad). All error bars show the standard deviation.

## Supplementary information

Supplemental movie 1

Supplemental movie 2

Supplementary Figure 1

Supplementary Figure 2

Supplementary data

Original data

Supplementary Material

## Data Availability

The authors confirm that the data supporting the findings of this study are available within the article [and/or] its supplementary materials.
