# Supplementary figures and images for: The chaperonin TRiC component Cct3 is required for axonal transport, myelination, and neuromuscular junction refinement
Source: Cell Death Dis. 2026 Feb 12;17(1):221. doi: 10.1038/s41419-026-08465-y (PMC12921322; doi:10.1038/s41419-026-08465-y)

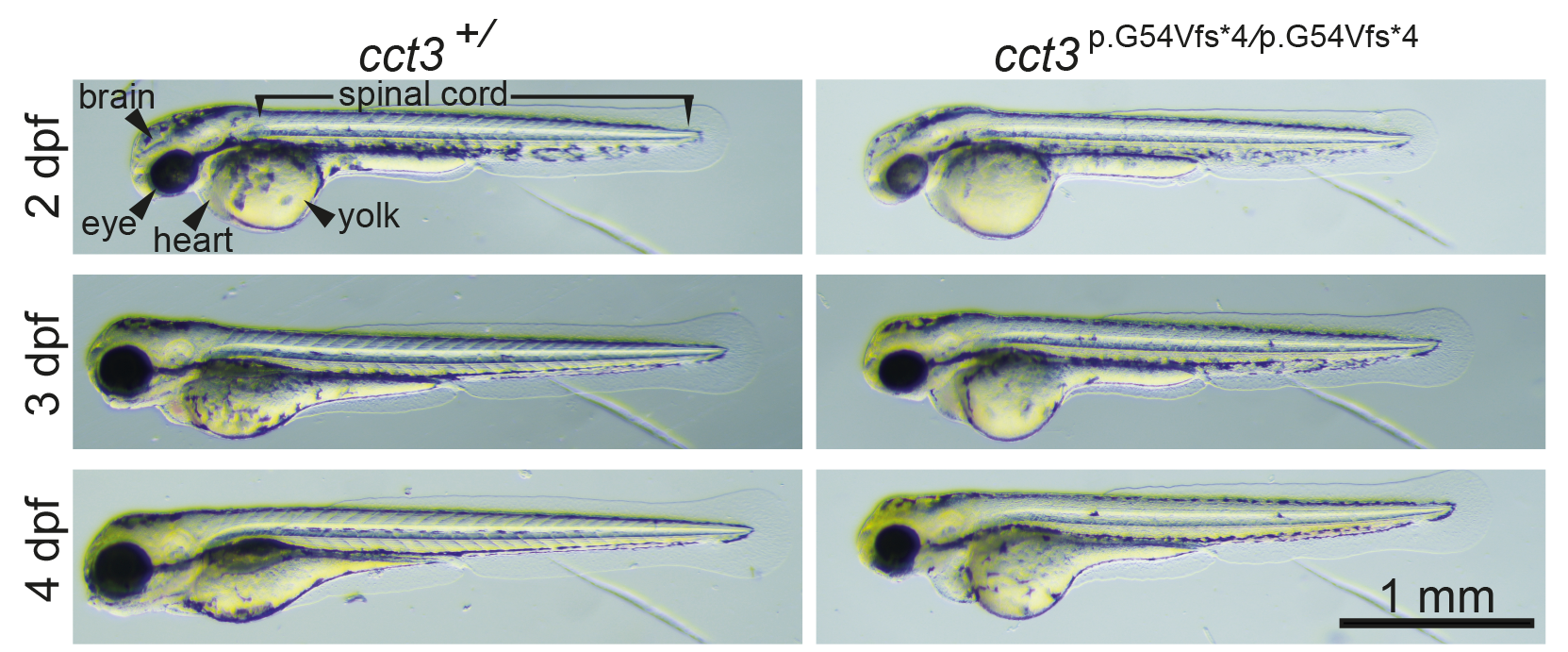

Supplement: Supplementary file 3 — Supplementary Figure 1 [file 41419_2026_8465_MOESM3_ESM.tif]

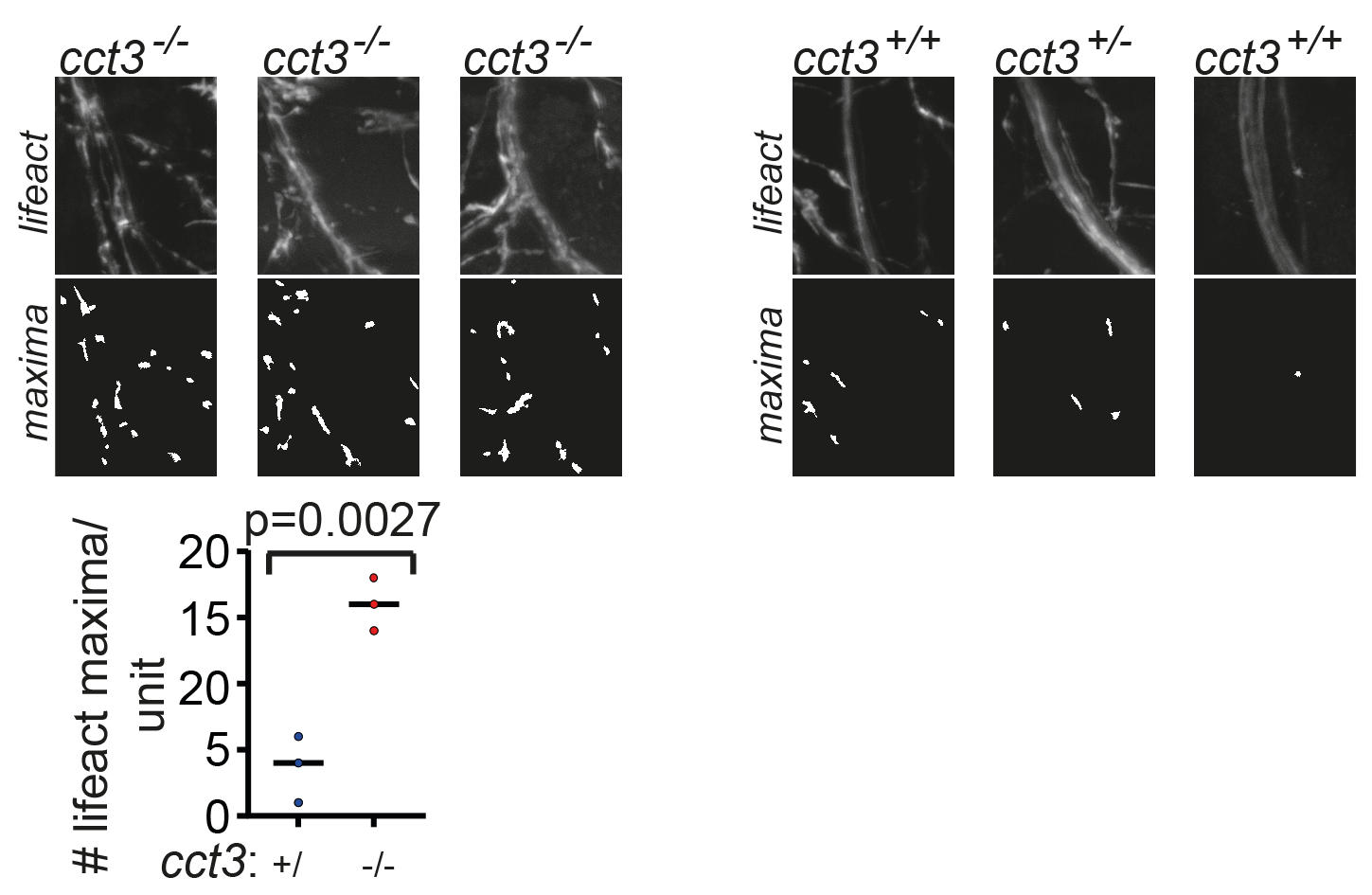

Supplement: Supplementary file 4 — Supplementary Figure 2 [file 41419_2026_8465_MOESM4_ESM.tif]

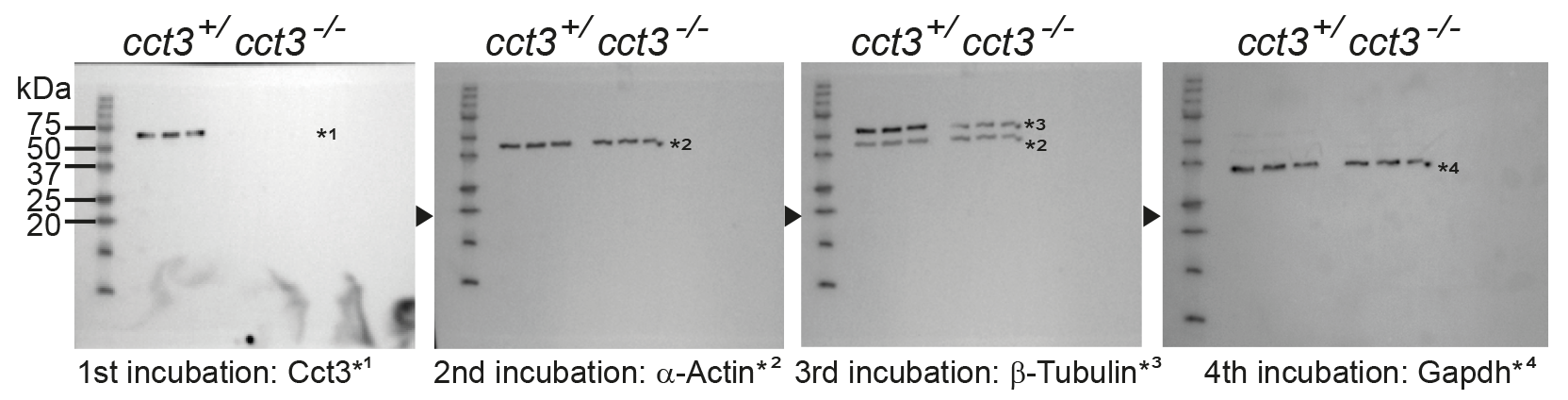

Supplement: Supplementary file 6 — Original data [file 41419_2026_8465_MOESM6_ESM.tif]
