# Supplementary material for: The chaperonin TRiC component Cct3 is required for axonal transport, myelination, and neuromuscular junction refinement
Source: Cell Death Dis. 2026 Feb 12;17(1):221. doi: 10.1038/s41419-026-08465-y (PMC12921322; doi:10.1038/s41419-026-08465-y)
Supplement: Supplementary file 7 — Supplementary Material [file 41419_2026_8465_MOESM7_ESM.docx]

**Supplementary Material**

**Supplementary Movie 1**

Movie showing the motoneuronal axonal mitochondrial distribution and transport. They were labeled by the combination of *Tg(mnx1:Gal4)* and *Tg(UAS:mito-mCherry).* Timelpase with 4 s intervals are shown of 4 wt siblings and 4 mutant nerves.

**Supplementary Movie 2**

Movie showing the motoneuronal axonal Rab7+ endosome distribution and transport. They were labeled by the combination of *Tg(mnx1:Gal4)* and *Tg(UAS:EGFP-Rab7).* Timelpase with 4 s intervals are shown of 4 wt siblings and 4 mutant nerves.

**Supplementary Figure 1. Further phenotypic data on *cct3* mutants.** Similar to the compound heterozygous mutants displayed in Figure 1, p.G54Vfs*4 mutant larvae at 2-4 dpf are smaller in overall size, in particular brain and eyes are smaller. This difference gets more pronounced with age. On day 4, mutant larvae also display cardial edema.

**Supplementary Figure 2. F-actin irregularities in *cct3* mutant inner axons.** To assess F-actin irregularities in the lifeact-transgenic line (see Figure 5A), a region of the inner nerve stump just below the horizontal myoseptum was selected and processed in FIJI to find maxima with a prominence of >50.00. Maxima within tolerance were counted and are displayed. P-values were determined using the two-tailed unpaired Student’s t-test.

**Supplementary data file**

Data used to generate the graphs are summarized, including information about genotypes and statistical test to compare wt and heterozygous siblings where applicable.

**Supplementary Original data. Full length Western blot data.** Uncropped membranes of the Western blot data in Figure 1 and Figure 5 are shown. Membranes were incubated with the indicated primary antibodies (each time followed by the appropriate secondary antibody) in the indicated order (from left to right). Signals are marked by * with the appropriate number. Note that after incubation with the α-tubulin antibody, the β-actin antibody labeling was still detectable.
